# Effects of different inspiratory muscle training protocols on functional exercise capacity and respiratory and peripheral muscle strength in patients with chronic kidney disease: a randomized study

**DOI:** 10.1186/s12882-024-03610-1

**Published:** 2024-05-29

**Authors:** Nihan Katayıfçı, İrem Hüzmeli̇, Döndü İri̇ş, Faruk Hilmi Turgut

**Affiliations:** 1 Department of Physiotherapy and Rehabilitation, Faculty of Health Sciences, Hatay Mustafa Kemal University, Hatay, Turkey; 2 Department of Physiotherapy and Rehabilitation, Institute of Health Sciences,, Hatay Mustafa Kemal University, Hatay, Turkey; 3 Department of Nephrology, Tayfur Ata Sokmen Faculty of Medicine, Hatay Mustafa Kemal University, Hatay, Turkey

**Keywords:** Chronic kidney disease, Respiratory muscle training, Exercise test, Muscle strength, Dyspnoea

## Abstract

**Background:**

Pathological changes were observed in the diaphragm due to abnormal renal function in chronic kidney disease (CKD). Inspiratory muscle training (IMT) has been suggested for patients with CKD; however, the most appropriate intensity for IMT has not been determined. Therefore, this study aimed to investigate the effects of different IMT protocols on respiratory muscle strength, quadriceps femoris muscle strength (QMS), handgrip muscle strength (HGS), functional exercise capacity, quality of life (QoL), pulmonary function, dyspnoea, fatigue, balance, and physical activity (PA) levels in patients with CKD**.**

**Methods:**

This randomized, controlled, single-blind study included 47 patients and they were divided into three groups: Group 1 (*n* = 15, IMT with 10% maximal inspiratory pressure (MIP)), Group 2 (*n* = 16, IMT with 30% MIP), and Group 3(*n* = 16; IMT with 60% MIP). MIP, maximal expiratory pressure (MEP), 6-min walking test (6-MWT), QMS, HGS, QoL, pulmonary function, dyspnoea, fatigue, balance, and PA levels were assessed before and after eight weeks of IMT.

**Results:**

Increases in MIP, %MIP, 6-MWT distance, and %6-MWT were significantly higher in Groups 2 and 3 than in Group 1 after IMT (*p* < 0.05). MEP, %MEP, FEF_25-75%,_ QMS, HGS, and QoL significantly increased; dyspnoea and fatigue decreased in all groups (*p* < 0.05). FVC, PEF, and PA improved only in Group 2, and balance improved in Groups 1 and 2 (*p* < 0.05).

**Conclusions:**

IMT with 30% and 60% MIP similarly improves inspiratory muscle strength and functional exercise capacity. IMT with 30% is more effective in increasing PA. IMT is a beneficial method to enhance peripheral and expiratory muscle strength, respiratory function, QoL and balance, and reduce dyspnoea and fatigue. IMT with %30 could be an option for patients with CKD who do not tolerate higher intensities.

**Trial registration:**

This study was retrospectively registered (NCT06401135, 06/05/2024).

## Introduction

Chronic kidney disease (CKD) is recognized as a global health problem, affecting approximately 11–13% of the world's population, and is associated with high economic costs to healthcare systems [1]. In addition, patients with CKD have a high risk of cardiovascular mortality [2]. Exercise intolerance, one of the most important risk factors for cardiac mortality, was found to be lower in patients with CKD than in healthy individuals [3]. Exercise training, which improves functional capacity and quality of life (QoL), has been recommended as an essential element in the treatment of patients with CKD [4]. Furthermore, respiratory muscle weakness has been reported in patients with CKD [5]. Management of respiratory muscle weakness is crucial, as reduced functional performance is related to respiratory muscle weakness.

Inspiratory muscle training (IMT) is recommended for people with CKD pre-dialysis period and on dialysis to increase respiratory muscle strength, exercise capacity, respiratory, and QoL. Different IMT protocols with 30–70% of the maximal inspiratory pressure (MIP) have been found to improve these in patients with CKD [6–8]. Studies investigating the effect of IMT have mostly focused patients on dialysis [7]; however, patients with CKD had respiratory muscle weakness before the pre-dialysis period [9]. Decrease in respiratory and peripheral muscle strength, pulmonary function, and exercise capacity; increase in dyspnea was shown in patients with early stages [9, 10]. Therefore, it is necessary to pay attention to the effect of IMT on patients with CKD who are not on dialysis. Previous studies have investigated the effects of IMT in patients with CKD, and these studies aimed to investigate the effects of IMT varying from 30–70% of MIP in patients with CKD compared with a control group that had no resistance or very low intensity [6, 7]. The most efficient IMT intensity for patients with CKD is unknown, therefore, this study aimed to investigate the effects of different IMT protocols on respiratory muscle strength, functional exercise capacity, quadriceps femoris muscle strength (QMS), handgrip muscle strength (HGS), QoL, respiratory function, dyspnoea, fatigue, balance, and PA levels in patients with CKD that were not on dialysis.

## Material and methods

### Study design

This was a prospective, randomized, controlled, single-blind study. The study was approved by the Ethics Committee of Hatay Mustafa Kemal University (No.2021/10). Written informed consent was obtained from all patients included in the study (ClinicalTrials number: NCT06401135, Date: 06/05/2024). Patients were randomized by using computer-based block randomization to the following groups: Group 1 (10% MIP), Group 2 (30% MIP), or Group 3 (60% MIP). The study director put the allocation sequences in a sealed opaque envelope until group allocation. A research assistant, independent from the study's researchers, allocated patients to groups by sequentially opening each envelope as they entered the study. Patients were assigned to groups based on the predetermined order within the envelopes. The primary outcome was inspiratory muscle strength (IMS). The secondary outcomes were, functional exercise capacity QMS, HGS and expiratory muscle strength (EMS), pulmonary function, dyspnoea, fatigue, QoL, PA, and balance.

### Patients

Forty-seven patients with CKD who were referred to the department between 28 July 2021 and 30 October 2022 were included. The inclusion criteria were patients with CKD stages 1–5 based on the CKD staging proposed by the Kidney Disease Outcomes Quality Initiative [11] who were not on dialysis aged > 18 years and clinically stable. The exclusion criteria were patients with CKD who have uncontrolled hypertension, non-stable cardiac disease, recent viral infections, respiratory, neurological, and orthopedic diseases, or a history of malignancy.

### Measurements

A mouth pressure device (Micro Medical MicroRPM, England) was used to evaluate IMS and EMS (MEP). MIP and MEP were expressed as actual values and as a percentage of expected values [12, 13]. Values < 80% of the predicted MIP and MEP were used to identify respiratory muscle weakness. The minimal clinically important difference (MCID) was 11 cmH2O for MIP [14].

According to American Thoracic Society/ European Respiratory Society guidelines, spirometry was used to assess pulmonary function (Spirobank MIR, Rome, Italy). Percentages of forced vital capacity (FVC), forced expiratory volume in the first second (FEV_1_), expiratory peak flow (PEF), forced expiratory flow from 25–75% (FEF_25-75(%)_), and the FEV_1_/FVC ratio were used [15].

A hand-held dynamometer (JTECH Power Track Commander, Baltimore, MD, USA) was used to measure QMS. The percentage of predicted values was calculated according to the reference values [16]. Hand-grip strength was assessed using a Jamar analog hand dynamometer (PowerTrack II; JTECH Medical, Midvale, Utah, USA) [17]. The measurements were repeated three times, and the highest value was used for comparison.

The 6-MWT, which measures functional exercise capacity, was performed in an enclosed 30-m corridor. The patients walked as fast as they could within 6 min. Patients performed the test twice and rested for at least 30 min between the tests [18]. The 6-MWT distance was expressed as actual values and as a percentage of expected values [19]. The MCID is 66.3 m for the 6-MWT in CKD [20].

Dyspnoea during activity was measured using the Modified Medical Research Council (MMRC) dyspnoea scale [21]. Levels of dyspnea were graded 0–4. Fatigue was assessed using the Fatigue Severity Scale (FSS). The total score ranges from 0 to 7. Severe fatigue was detected in patients with scores ≥ 4 [22].

The Short Form 36 (SF-36) questionnaire was used to assess QoL and includes both physical and mental components. The scores range from 0 to 100, with higher values indicating better health [23].

Physical activity was identified using the International Physical Activity Questionnaire (IPAQ) short form, which included questions on sitting duration, walking activity, and moderate and vigorous activities. The IPAQ is categorized as inactive (< 600 MET-min/week), minimally active (600–3000 MET-min/week), and sufficiently active (> 3000 MET-min/week) based on total scores [24].

Balance was evaluated using the Berg Balance Scale (BBS). The scale includes 14 items scoring 0 to 4. Higher scores indicate better balance [25].

### Training program

IMT was conducted using a pressure threshold-loading device (POWERbreathe® Classic Low Resistance). Group 1 performed IMT at 10% of MIP, Group 2 at 30% of MIP, and Group 3 at 60% of MIP. The MIP values of all patients in all groups were measured during supervised sessions each week. The new training workload was determined by new MIP values. The patients underwent IMT seven days per week for a total of eight weeks. IMT sessions were performed in six sessions at home, and one under supervision. During the 30-min IMT session, patients maintained diaphragmatic breathing with 10–15 breaths followed by 5–10 s of rest, and vital signs were monitored during the sessions. Patients were given a diary for their IMT periods to record IMT sessions and adverse effects during sessions. The patients were not informed about their groups and they were evaluated and trained at different places and times. The assessments and interventions were carried out by the same physiotherapist.

### Statistical analysis

Data were analyzed using the SPSS 20.0 statistical analysis program (Armonk, NY, IBM Corp). The G*Power software program was used to estimate the sample size. In a previous study, MIP results (ES: 0.34), a sample size estimation with 80% power (α = 0.05), and an effect size of 0.733 were performed, and 39 patients were calculated [26]. Considering a potential dropout rate of 20%, 47 patients were enrolled in the study. Shapiro–Wilk test was used to test data normality. Descriptive characteristics were presented as percentages for qualitative data and as mean (± standard deviation) or median (IQR) for quantitative data. Baseline characteristics of the three groups were compared using analysis of variance (ANOVA) and the Kruskal–Wallis test. Nominal data were compared using the Chi-square test. The effects of the interventions were compared using Repeated Measures ANOVA (within-group, between-group, and timed group interactions) with Tukey’s post hoc test. The effects of interventions of the groups over time were analyzed using the Mann Whitney U/ Independent samples t-test as pairwise comparisons. Statistical significance was set at *p* < 0.05. Effect size (ES) calculated to partial eta square (η2). In accordance with the literature, an ES of η2 = 0.01 indicated a small effect, η2 = 0.06 indicated a medium effect and η2 = 0.14 indicated a large effect [27, 28].

## Results

**Fig. 1:**
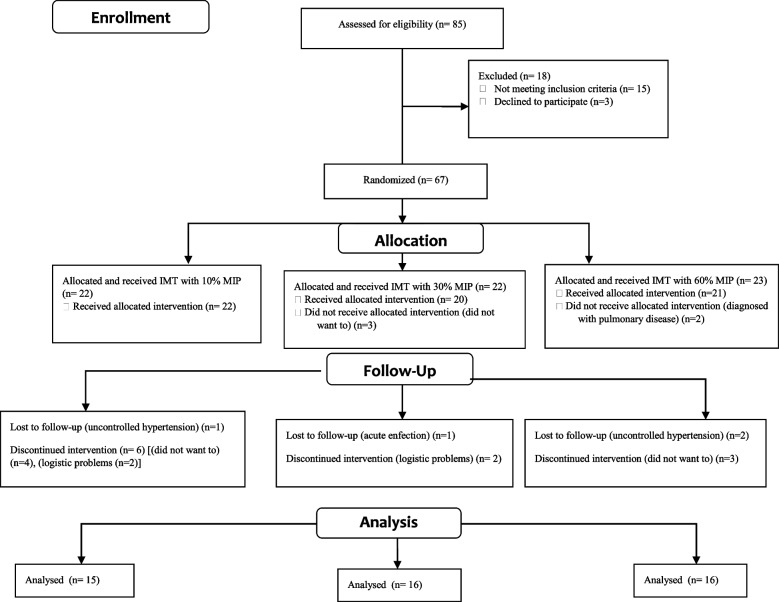
CONSORT Flow diagram of recipients

Between July 2021 and October 2022, 85 patients with CKD were directed for the study. Eighteen patients were excluded for various reasons. Sixty-seven patients were randomly assigned to Groups 1, 2, or 3. At least 47 patients completed the study (Fig. 1). Baseline characteristics were statistically similar between the three groups (*p* > 0.05), except for PEF % (*p* = 0.019) (Tables 1, 2).

**Table 1:** Demographic characteristics of patients with CKD

Variables	Group I X ± SD/ Median (IQR)	Group II X ± SD/ Median (IQR)	Group III X ± SD/ Median (IQR)	*p*
Age (years)	57 (37–65)	63 (51.25–67.50)	55.50 (41.25–58.50)	0.127
Weight (kg)	73.85 ± 18.46	86.29 ± 15.87	76.13 ± 14.03	0.083
Height (cm)	163.66 ± 8.19	170.12 ± 6.96	167.81 ± 7.33	0.064
Body mass index (kg/m^2^)	27.49 ± 6.24	29.57 ± 5.61	26.91 ± 4.36	0.357
Female, Male n (%)	5/33.3%, 10/66.7%	3/18.8%, 13/81.3%	5/31.3%, 11/68.8%	0.613
Smoking (current/ex/non-smoker), *n* (%)	2/13.3%, 6/40%, 7/46.7%	4/25%, 3/18.8%, 9/56.3%	3/18.8%, 4/25%, 9/56.3%	0.726
Comorbidities				
Hypertension, *n* (%)	8/53.3%	11/68.8%	10/62.5%	0.675
Diabetes mellitus, *n* (%)	6/40%	6/37.5%	3/18.8%	0.376
Coronary artery disease, *n* (%)	4/26.7%	0/0%	2/12.5%	NA
CKD stages (1–5), *n* (%)	1/6.7%, 2/13.3%, 7/46.7%, 1/6.7%, 4/26.7%	1/6.3%, 2/12.5%, 6/37.5%, 4/25.0%, 3/18.8%	2/12.5%, 2/12.5%, 6/37.5%, 4/25%, 2/12.5%	0.916
MIP weakness (< 80%, > 80% of the predicted values), *n* (%)	8/53.3%, 7/46.7%	10/62.5%, 6/37.5%	2/12.5%, 14/87.5%	**0.010**
MEP weakness (< 80%, > 80% of the predicted values), *n* (%)	12/80%, 3/20%	12/75%, 4/25%	8/50%, 8/50%	0.154

*NA* Not analyzed, *CKD* Chronic kidney disease, *MIP* Maximal inspiratory pressure, *MEP* Maximal expiratory pressure

*p* < 0.05

**Table 2:** Effects of different inspiratory training protocols on respiratory muscle strength, exercise capacity, pulmonary functions, peripheral muscle strength, quality of life, dyspnea, fatigue, physical activity level and balance

	**Group I** **X ± SD**			**Group II** **X ± SD**			**Group III** **X ± SD**			**Treatment effect**
Variables	**Before**	**After**	**Group difference** ***p***	**Before**	**After**	**Group difference** ***p***	**Before**	**After**	**Group difference** ***p***	***p***
MIP (cmH_2_O)	**75.53 ± 23.16**	**94.61 ± 22.16**	** < 0.001**	**71.12 ± 26.16**	**104.29 ± 24.90**	** < 0.001**	**80.81 ± 22.37**	**111.76 ± 24.19**	** < 0.001**	**0.006**
%MIP	**83.04 ± 27.99**	**104.11 ± 28.90**	** < 0.001**	**78.13 ± 26.63**	**115.08 ± 24.46**	** < 0.001**	**87.21 ± 21.37**	**121.15 ± 23.19**	** < 0.001**	**0.004**
MEP (cmH_2_O)	**76.0 ± 22.53**	**89.25 ± 20.35**	** < 0.001**	**89.06 ± 34.36**	**108.49 ± 34.73**	** < 0.001**	**90.12 ± 36.72**	**108.36 ± 38.66**	** < 0.001**	0.288
%MEP	**64.89 ± 15.54**	**76.52 ± 12.91**	** < 0.001**	**78.25 ± 31.16**	**94.80 ± 30.97**	** < 0.001**	**76.27 ± 27.12**	**92.22 ± 28.62**	** < 0.001**	0.263
6MWT (m)	**443.81 ± 84.53**	**489.06 ± 74.12**	** < 0.001**	**408.41 ± 129.68**	**488.99 ± 129.01**	** < 0.001**	**457.16 ± 105.02**	**530.84 ± 106.74**	** < 0.001**	**0.018**
%6MWT	**72.55 ± 13.58**	**80.31 ± 14.29**	** < 0.001**	**70.59 ± 18.25**	**84.98 ± 18.14**	** < 0.001**	**72.88 ± 18.95**	**84.39 ± 18.74**	** < 0.001**	**0.020**
FEV_1_ (%)	84.66 ± 15.80	87.87 ± 14.57	0.178	**79.28 ± 20.03**	**87.90 ± 20.90**	**0.001**	86.81 ± 18.57	89.07 ± 19.25	0.324	0.138
FVC (%)	87.86 ± 17.18	87.23 ± 14.71	0.809	**77.46 ± 20.64**	**87.38 ± 16.15**	** < 0.001**	89.13 ± 18.80	90.91 ± 21.75	0.646	**0.013**
FEV_1_/FVC	84.02 ± 10.81	84.29 ± 10.18	0.860	80.34 ± 13.23	80.19 ± 13.95	0.922	85.38 ± 19.75	85.61 ± 20.18	0.879	0.977
PEF (%)	68.50 ± 20.06	67.58 ± 18.70	0.826	**51.23 ± 12.22**	**70.77 ± 19.33**	** < 0.001**	66.13 ± 15.15	70.93 ± 17.63	0.273	**0.005**
FEF_%25–75_ (%)	**73.53 ± 26.31**	**83.76 ± 24.99**	**0.013**	**73.13 ± 30.85**	**89.81 ± 31.21**	** < 0.001**	**80.87 ± 28.31**	**91.47 ± 28.45**	**0.008**	0.433
Quadriceps femoris, (Dom)	**197.78 ± 62.69**	**246.27 ± 68.40**	** < 0.001**	**207.58 ± 85.53**	**258.98 ± 68.62**	** < 0.001**	**178.60 ± 70.83**	**239.16 ± 65.76**	** < 0.001**	0.680
Handgrip, (Dom), P	**65.20 ± 24.15**	**71.69 ± 24.79**	** < 0.001**	**72.56 ± 27.26**	**79.02 ± 27.16**	** < 0.001**	**72.18 ± 25.50**	**80.81 ± 26.30**	** < 0.001**	0.456
MMRC (0–4)	**1.0 ± 0.92**	**0.40 ± 0.63**	** < 0.001**	**1.43 ± 1.15**	**0.62 ± 0.80**	** < 0.001**	**1.18 ± 0.65**	**0.50 ± 0.63**	** < 0.001**	0.512
FSS score (0–7)	**3.91 ± 1.98**	**2.56 ± 2.04**	** < 0.001**	**3.85 ± 2.60**	**2.64 ± 2.19**	** < 0.001**	**2.56 ± 2.24**	**1.78 ± 1.85**	**0.001**	0.159
IPAQ										
Total PA (MET, min./week)	652.30 ± 608.38	909.33 ± 744.40	0.847	**772.82 ± 1700.0**	**3708.42 ± 10,775.35**	**0.039**	798.80 ± 1019.57	1076.48 ± 1021.87	0.835	0.288
Vigorous physical activity (MET, min./week)	0	53.33 ± 160.0	0.975	201.60 ± 637.51	2878.65 ± 8445.46	0.104	0	160.0 ± 391.91	0.938	0.462
Moderate physical activity (MET, min./week) 26.66 ±	26.66 ± 52.91	11.11 ± 22.60	0.980	420.80 ± 1327.87	1473.60 ± 4218.10	0.086	160.0 ± 290.65	160.0 ± 290.65	1.000	0.389
Walking activity (MET, min./week)	900.16 ± 662.83	1127.13 ± 874.42	0.276	268.95 ± 308.20	565.95 ± 802.39	0.138	1125.25 ± 1550.68	1191.13 ± 1513.84	0.793	0.840
Sitting duration, min	397.50 ± 192.26	405.0 ± 197.04	0.905	**470.0 ± 218.40**	**306.66 ± 151.32**	**0.011**	365.0 ± 76.87	290.0 ± 148.99	0.307	0.159
SF-36 subscales (0–100)										
PF	**72.14 ± 17.39**	**80.97 ± 15.20**	**0.012**	**72.69 ± 26.26**	**82.49 ± 23.91**	**0.008**	85.66 ± 15.79	90.32 ± 14.25	0.160	0.516
PRF	66.07 ± 45.58	65.88 ± 39.85	0.986	**50.00 ± 44.48**	**64.03 ± 43.18**	**0.038**	**66.66 ± 41.90**	**73.65 ± 38.23**	**0.011**	0.663
ERF	66.66 ± 47.14	70.03 ± 42.89	0.705	69.23 ± 48.03	74.71 ± 42.71	0.553	68.89 ± 44.48	74.26 ± 39.00	0.532	0.982
VT	**53.57 ± 23.81**	**63.01 ± 18.41**	**0.027**	**57.30 ± 26.03**	**73.64 ± 24.84**	** < 0.001**	69.00 ± 21.31	76.97 ± 18.34	0.051	0.328
MH	**68.00 ± 19.40**	**78.01 ± 16.35**	**0.014**	66.46 ± 23.69	74.47 ± 23.29	0.054	68.93 ± 18.52	74.68 ± 18.67	0.134	0.734
SF	68.75 ± 25.35	66.31 ± 20.16	0.659	75.00 ± 21.04	83.10 ± 27.32	0.161	79.56 ± 20.94	81.89 ± 21.42	0.661	0.417
BP	76.78 ± 27.14	75.85 ± 22.92	0.805	**65.57 ± 28.15**	**78.59 ± 19.28**	**0.002**	84.53 ± 22.14	80.87 ± 23.94	0.318	**0.008**
GH	58.92 ± 13.47	58.95 ± 13.18	0.995	55.38 ± 27.34	57.32 ± 24.55	0.667	60.11 ± 20.55	63.90 ± 18.21	0.585	0.786
Physical health	68.48 ± 19.31	70.41 ± 15.52	0.621	**60.91 ± 24.55**	**70.61 ± 23.19**	**0.021**	75.50 ± 19.67	76.97 ± 18.99	0.706	0.264
Mental health	64.24 ± 21.0	69.34 ± 17.84	0.193	**67.0 ± 25.41**	**76.48 ± 27.34**	**0.023**	71.59 ± 22.25	76.95 ± 20.50	0.158	0.678
BBS score (0–56)	**53.15 ± 3.73**	**54.69 ± 3.27**	**0.050**	**50.15 ± 5.20**	**53.84 ± 3.55**	** < 0.001**	55.0 ± 1.19	55.60 ± 0.82	0.402	**0.017**

*FEV*_*1*_ Forced expiratory volume in 1 s, *FVC* Forced vital capacity, *PEF* Peak expiratory flow, *FEF*_*%25–75*_* (%)* Forced expiratory flow at 25–75% of the pulmonary volume, *6MWT* 6 min walk test, *MIP* Maximal inspiratory pressure, *MEP* Maximal expiratory pressure, *ND* Non-dominant, *MMRC* Modified Medical Research Council, *FSS* Fatigue Severity Scale, *IPAQ* International Physical Activity Questionnaire, *PA* Physical activity, *BBS* Berg Balance Scale, *PF* Physical functioning, *PRF* Physical role functioning, *ERF* Emotional role functioning, *VT* Vitality, *MH* Mental health, *SF* Social functioning, *BP* Bodily pain, *GH* General health, *NA* Not analyzed

*p* < 0.05

**Fig. 2:**
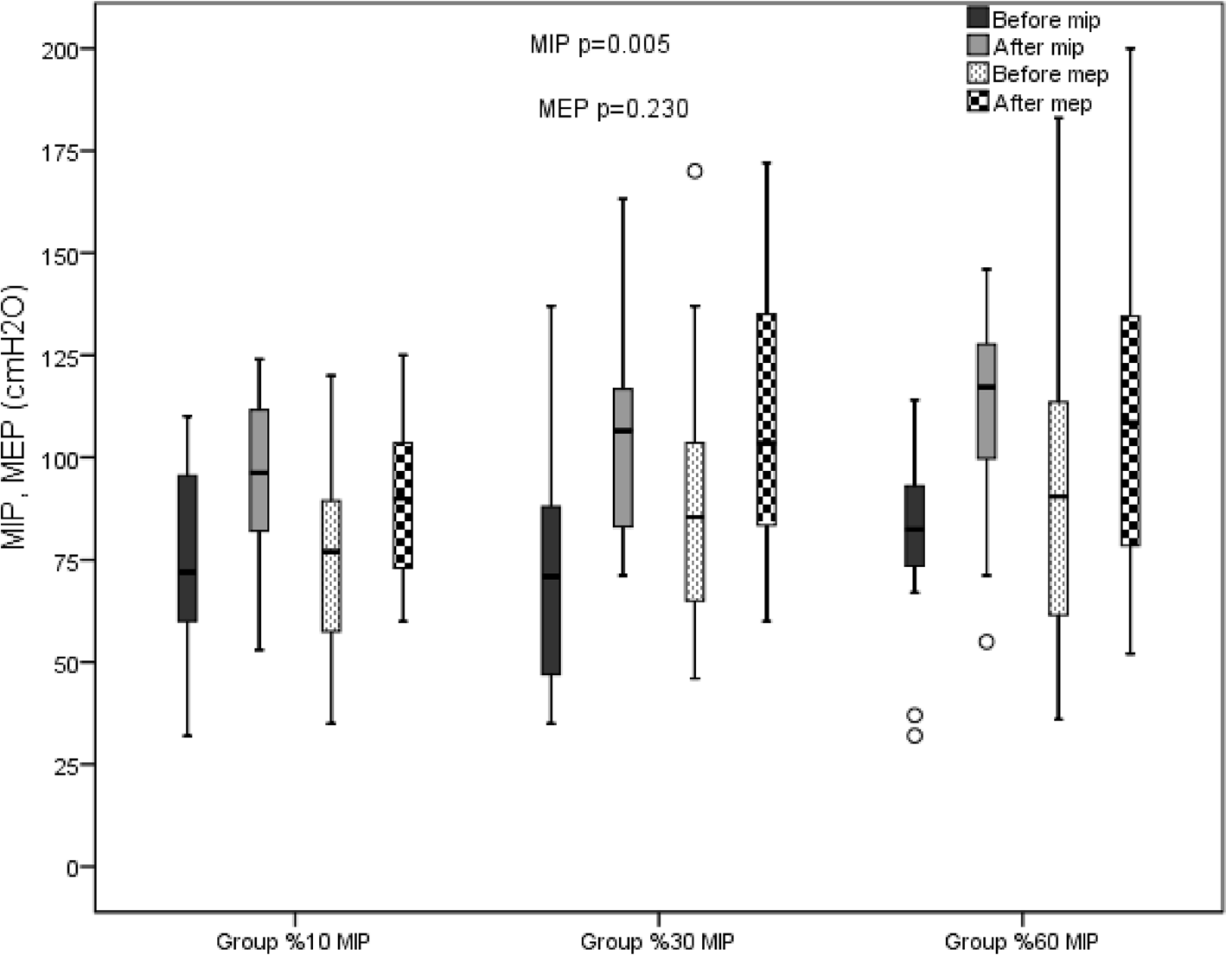
MIP and MEP values before and after in Group 1, 2 and 3

MIP (ES:0.21) and %MIP (ES:0.21) were significantly increased both in and between groups (*p* < 0.05, Table 2, Fig. 2). The increase in MIP after IMT was statistically higher in Groups 2 (*p* < 0.001) and 3 (*p* = 0.002) than Group 1 (∆MIP 19.08 cmH2O, 95%CI = 12.65–25.51 cmH2O in Group 1, ∆MIP 33.16 cmH2O, 95%CI = 26.93–39.39 cmH2O in Group 2, and ∆MIP 30.95 cmH2O, 95%CI = 24.72–37.18 cmH2O in Group 3). Improvement in MIP was over MCID in all patients in Groups 2 and 3, and 86.7% of the patients in Group 1. MEP and %MEP significantly improved in all groups (*p* < 0.05); however, there were no significant differences between the groups (*p* > 0.05, Table 2, Fig. 2).

A significant difference was found in the 6-MWT distance (ES:0.17) and %6-MWT (ES:0.16) between the groups (*p* < 0.05, Table 2). The increase in 6-MWT distance was statistically higher in Groups 2 (*p* = 0.012) and 3 (*p* = 0.006) than Group 1 (Group 1 [∆6-MWT distance 45.25 m, 95%CI = 27.30–63.20 m, %∆6-MWT 7.75%, 95%CI = 4.52–10.98%], Group 2 [∆6-MWT 80.57 m, 95%CI = 62.62–98.52 m, %∆6-MWT 14.39%, 95%CI = 11.15–17.62%], and Group 3 [∆6-MWT 73.68 m, 95%CI = 56.03–91.06 m, %∆6-MWT 11.51%, 95%CI = 8.38–14.64%]) after IMT (*p* < 0.05, Table 2). Of the patients, 26.7% in Group 1, 68.8% in Group 2, and 75% in Group 3 reached MCID (66.3 m) at the 6-MWT distance. There was no significant improvement in FEV_1_/FVC within and between groups (*p* > 0.05). FEV_1_%, FVC%, and PEF% were significantly increased in Group 2, and FEF_25-75(%)_ was significantly increased in all groups (*p* < 0.05, Table 2). While there were no significant differences in FEV_1_% and FEF_25-75(%)_ between groups (*p* > 0.05, Table 2), there was a significant difference in FVC% (ES:0.18) and PEF% (ES:0.25) between the groups (*p* < 0.05, Table 2).

The QMS and percentage and HGS were significantly increased, and the MMRC and FSS scores were decreased in all groups (*p* < 0.05); however, there were no significant differences between the groups (*p* > 0.05, Table 2). The IPAQ total PA, SF-36 physical and mental health, and bodily pain sub-parameter scores were increased significantly only in Group 2 (*p* < 0.05). Physical functioning and vitality sub-parameter scores were increased in Group 1 and 2 (*p* < 0.05). Physical role functioning sub-parameter scores were increased in Group 2 and 3 (*p* < 0.05). Mental health sub-parameter scores were increased significantly only in Group 1 (*p* < 0.05). IPAQ vigorous and moderate PA and walking scores did not differ significantly in and between groups (*p* < 0.05, Table 2). The IPAQ sitting duration was significantly decreased only in Group 2 (*p* < 0.05, Table 2). The BBS score significantly increased in Groups 1 and 2 (*p* < 0.05, Table 2, ES:0.19). None of the patients experienced adverse effects during the IMT sessions. Patients in Group 1 attended 91%, Group 2 attended 89% and Group 3 attended 88% of IMT sessions.

## Discussion

The current study was the first to investigate different training protocols. The present study found that IMT with 30 and 60% of MIP similarly increases functional exercise capacity; the highest improvement in PA is seen in IMT with 30%; IMT increases QMS, HGS, MEP, respiratory functions, and balance, reduces dyspnoea and fatigue.

Different training protocols that varied from 40 to 70% were used for IMT in patients with CKD [6, 7, 29–32]. Both IMT studies with 50–70% MIP [7, 29, 32] and 40% MIP [30, 33] showed improvement in IMS. In addition, studies with IMT with 40% MIP (25.92 cmH2O) [30], IMT with 50% MIP (22.5 cmH2O) [29], and IMT with 70% MIP (23.4 cmH2O) [7] showed an increase in MIP compared with controls that were no resistance, very low resistance, or no training. In the current study, MIP improved in all three groups, but IMT with 30% (33.16 cmH2O) and 60% (30.95 cmH2O) MIP showed higher improvements than IMT with 10% MIP (19.08 cmH2O). A minimal load of 30% MIP was found to improve in patients with chronic obstructive pulmonary disease [34]. However, in the present study, the IMT group with 10% MIP showed an improvement. Medeiros et al., stated an improvement in MIP in both training (50% MIP) and control (minimum device load; 5 cmH2O) groups in patients with CKD hemodialysis [32]. Owing to different disease mechanisms, the effects of IMT might be diverse in patients with CKD [32, 34]. IMT with 30% MIP may be an effective protocol for patients with CKD who cannot tolerate high intensities.

Studies about the effects of IMT on MEP in patients with CKD are limited. Three studies showed an increase in MEP [29, 32, 33], two 50% MIP studies showed that improvement varies from 10.8 to 23.16 cmH2O [29, 32], and only one 40% MIP study found an increase in MEP (73.12 to 82.50 cmH2O) [33]. Although there was no significant difference, the highest increase was observed in the group treated with 30% MIP. Of note, including patients with respiratory muscle weakness may have led to a greater increase in MEP than in the current study [32].

A decrease in exercise capacity has been reported in patients with CKD [35]. A systematic review reported an 80 m increase in the 6-MWT distance after IMT in patients with CKD, compared with controls [6]. However, in two studies with 50–70% MIP, there was no improvement in the 6-MWT distance [7, 32]. In the present study, the 6-MWT distance was improved in all groups, but the group with 30% MIP (Group 2: ∆6-MWT 80.57 m) and the group with 60% MIP (Group 3: ∆6-MWT 73.68 m) showed more improvement than the group with 10% MIP (Group 1: ∆6-MWT distance 45.25 m). In addition, the increase in the 6-MWT distance was higher than the MCID (66.3 m) [20]. Due to the short training period [7] and viral infection [32], there was no improvement in the 6-MWT distance in the abovementioned studies. It is known that mortality is related to reduced exercise capacity, [3] and so IMT might be beneficial for the survival of patients with CKD. Thus, the effects of IMT on mortality should be investigated in further studies.

Improvement in respiratory functions was shown in previous studies in patients with CKD [29, 30, 32, 33]; FVC was increased in studies with 40–50% MIP [29, 30, 33], and PEF improved in one study with 50% MIP [32]. In the current study, FEV_1_, FVC, and PEF were improved only in the 30% MIP group. FEF_25-75(%)_ was improved in all groups. To further investigate the effects of IMT, pulmonary function should be assessed in more detail, such as diffusion capacity.

The present study was the first to show that QMS and HGS improved after IMT in patients with CKD. In addition, 10, 30, and 60% MIP similarly improved QMS, and HGS. It was shown that IMT with 30% [36] 40% [37], 50%, and 70% [38] MIP improved QMS in patients with heart failure. In addition, it was known that IMT increased limb blood flow in patients with heart failure [39]. A reduction in muscle metaboreflex activity may be a factor in increased muscle strength [40], thus further studies are needed to identify the mechanisms underlying the effects of IMT on peripheral muscle strength in patients with CKD.

In the literature, only one IMT (40% MIP) study showed that dyspnoea was improved after eight weeks of IMT [30]; however, dyspnoea was reduced after IMT with 10, 30, and 60% MIP in the current study. A reduction in dyspnoea may be due to an improvement in exercise capacity and respiratory muscle strength, therefore, new studies are needed to investigate the effects of IMT on dyspnoea, as the presence of respiratory muscle weakness may have affected the results.

A reduction in fatigue after IMT was observed in only one study with a 50% MIP [29]; however, fatigue was assessed using a sub-scale of QoL assessment. In the present study, fatigue decreased in both groups with 10, 30, and 60% MIP. Improvements in respiratory and peripheral muscle strength may have led to reduced fatigue. In the literature, the effects of IMT on QoL are scarce in patients with CKD. Two studies after eight weeks of IMT with 40 and 50% MIP showed no differences [30, 32]. One IMT study with 50% MIP for ten weeks stated an improvement in QoL sub-scales of energy/fatigue, sleep, pain, and a list of symptoms/problems [29]. In the present study, the SF-36 physical and mental sub-scale scores were increased in the 30% MIP group. A recent systematic review/meta-analysis stated that the effect of respiratory muscle training on QoL was unclear in patients with CKD [8], thus the effects of different IMT training durations on QoL should be examined.

In the current study, total PA increased in all groups; however, the 30% MIP group showed a significant improvement in total PA. A study found that IMT with 30% and 50% similarly increased PA in patients with heart failure. They stated that an increase in PA could be due to patients feeling safe and a reduction in kinesiophobia [36]. In the current study, an increase in functional exercise capacity or patients feeling safe and confident [36] may be the reason for improvement in PA. The effects of IMT on kinesiophobia in patients with CKD should be investigated in future studies. Balance was improved in the 10 and 30% MIP groups after the IMT, and an increase in the QMS may improve balance. There is a high incidence of falls in patients with CKD [41], and IMT could be an option to prevent falls by improving balance.

### Limitations

This study had a few limitations. Although the 6-MWT is a valid and reliable test for assessing functional exercise capacity [18], cardiopulmonary exercise tests have not been performed because of technical problems. Balance was evaluated using the BBS [25], therefore, computer-based systems should be used in future studies. Although the control group performed 10% MIP, an improvement was observed in this group, which may be due to daily low-load training, therefore, the control group should be sham without load, in future studies.

## Conclusion

This is the first randomized, controlled study to indicate that IMT with 30% and 60% MIP similarly increases functional exercise capacity and IMS. The highest improvement in PA is shown in IMT with 30%. IMT increases QMS, HGS, and EMS, respiratory functions and balance, and reduces dyspnoea and fatigue. Taken together, IMT should be safely used in patients with CKD, and IMT with %30 could be an option for patients with CKD who do not tolerate higher intensities. IMT with 30% or 60% should be a safe and effective intervention in cardiopulmonary rehabilitation for patients with CKD not on dialysis. The effects of IMT on the abovementioned outcomes in different CKD stages should be investigated.

## Data Availability

The datasets used and/or analysed during the current study available from the corresponding author on reasonable request.
